# Typhus Group Rickettsiosis, Brazilian Amazon

**DOI:** 10.3201/eid2609.201305

**Published:** 2020-09

**Authors:** Antonio H.H. Minervino, Marcelo B. Labruna, Salatiel R. Dias, Francisco B. Costa, Thiago F. Martins, Phablo N.S. da Silva, Álvaro A. Faccini-Martínez

**Affiliations:** Federal University of Western Pará, Santarém, Brazil (A.H.H. Minervino, S.R. Dias, P.N.S. da Silva);; University of São Paulo, São Paulo, Brazil (M.B. Labruna, T.F. Martins);; Universidade Estadual do Maranhão, São Luís, Brazil (F.B. Costa);; Asociación Colombiana de Infectología, Bogotá, Colombia (Á.A. Faccini-Martínez)

**Keywords:** typhus, typhus group, rickettsiosis, rickettsial infections, rickettsia, bacteria, vector-borne infections, zoonoses, Brazilian Amazon

## Abstract

*Rickettsia rickettsii* infection is the only rickettsiosis included in the list of reportable diseases in Brazil, where typhus group rickettsioses, mainly murine typhus, have been underreported. We report a case of typhus group rickettsiosis with unique ecologic particularities in a patient from the Brazilian Amazon, where, to our knowledge, rickettsioses have not been reported.

Typhus group rickettsioses are vectorborne infectious diseases that include murine typhus, caused by *Rickettsia typhi*, and epidemic typhus, caused by *R. prowazekii* (*1*). *R. typhi* is maintained in an enzootic cycle involving small mammals (e.g., *Rattus* spp. rats and *Didelphis* spp. opossums) and their ectoparasites, mainly fleas (*2*). *R. typhi* is usually transmitted to humans by contamination of the bite site, mucosal or skin abrasions with rickettsia-containing ectoparasite feces, or inhalation in contaminated dust (*2*). *R. prowazekii* is transmitted mainly by feces of human clothing lice (*Pediculus humanus humanus*) or a sylvatic cycle in the United States by contact with ectoparasites of flying squirrels (*1*).

In Brazil, there have been few reports of murine typhus, mostly >60 years ago and all from human-modified landscapes in southeastern or southern regions, far from the Amazon (*3*). To our knowledge, Brazil has had only 1 case of recrudescent, epidemic typhus (Brill-Zinsser disease) in a refugee from Europe (*4*). Currently, Brazilian spotted fever (*R. rickettsii* infection), a tickborne disease, is the only rickettsiosis included in the list of notifiable diseases in Brazil (*3*). We report a new case of typhus group rickettsiosis in a patient from the Brazilian Amazon.

On August 27, 2019, a 37-year-old man was admitted to a hospital at Porto Trombetas District (Pará State, eastern Amazon region of Brazil) (Figure) because of 4 days of fever, chills, headache, malaise, and productive cough, associated with mild respiratory distress 72 hours after fever onset. His daily work consisted of outdoor herpetologic monitoring activities in Saracá-Taquera National Forest (35 km from Porto Trombetas) during the 2 weeks before disease onset. He reported that while in the forest, he removed an attached tick from his right thigh 10 days before disease onset.

**Figure:**
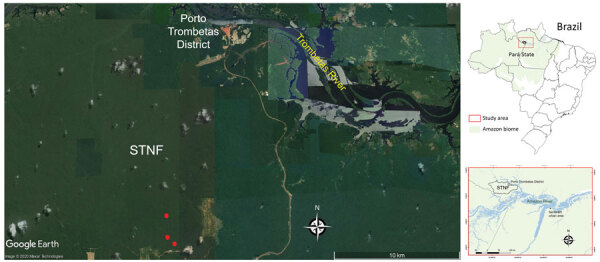
Study site in which typhus group rickettsiosis was detected in a 37-year-old man, Brazilian Amazon. Red dots indicate sites in STNF in which the patient worked during the day in the 2 weeks before disease onset. During this same period, he spent the night at his house in Porto Trombetas District, where he denied any rat infestation. Insets show location of STNF in Pará state and Brazil. STNF, Saracá-Taquera National Forest.

Physical examination showed fever (temperature 38.3°C), tachycardia, tachypnea, O_2_ saturation 91%, bilateral inguinal lymphadenopathy, no rash, and a furuncle lesion at the site of the tick bite on the right thigh, and no inoculation eschar. Blood analysis showed leukocytosis (15,000 cells/mm^3^); neutrophilia (84%); standard platelet count (221,000 platelets/μL); and increased levels of alanine aminotransferase (50 U/L), aspartate aminotransferase (38 U/L), and C-reactive protein (20 mg/L). Chest radiograph showed bilateral interstitial pulmonary infiltrates. Presumptive diagnoses of Lyme borreliosis or Brazilian spotted fever was made and the patient was admitted.

Supplementary oxygen, ceftriaxone, chloramphenicol, and doxycycline were administered, and serologic tests for *Borrelia burgdorferi* and *Rickettsia* spp. were performed. Twenty-four hours later, the patient showed clinical stability, major improvement of respiratory status, and no fever. After 7 days of antimicrobial drug treatment, he was discharged with complete resolution of symptoms.

A serum sample collected on August 28 (5 days after disease onset) showed negative results by commercial ELISA for *B. burgdorferi* IgM and IgG. Results of indirect fluorescent antibody assays were nonreactive for *R. conorii* IgG but positive (titer 1:64) for *R. typhi* IgG.

To confirm a presumptive diagnosis of rickettsiosis, we collected a second serum sample 2 months later and showed by using an in-house indirect fluorescent antibody assays (*5*) negative results for IgG (titer <1:64) against 6 *Rickettsia* species: *R. felis*; *R. bellii*; and the spotted fever group agents *R. rickettsii*, *R. parkeri*, *R. amblyommatis*, and *R. rhipicephali.* This serum sample had a titer of 1:2,048 against *R. typhi* (Wilmington strain), confirming typhus group rickettsiosis.

There have been previous reports of typhus group rickettsiae in ticks from other areas (*6**,**7*). After we considered the tick bite history of the patient, during October 12–14, 2019, we went to areas in Saracá-Taquera National Forest that the patient visited and collected 170 ticks in 7 species. Attempts to detect rickettsial DNA in these ticks showed only *R. amblyommatis* in *Amblyomma cajennense* sensu stricto ticks (Appendix).

In a retrospective interview, the patient recalled 3 additional activities during the 2 weeks before disease onset: a daily rest in the forest for a few minutes after work, seeking work materials in a small mammal trap-storage at a facility within the natural forest, and 2 short visits to his mother’s house in the urban area of Santarém municipality (Pará State). He denied rat infestation in his own house at Porto Trombetas but reported previous rat infestations in his mother’s house.

Borreliosis and rickettsioses have not been confirmed in the Brazilian Amazon (*8*). The patient’s tick bite history before onset of symptoms led clinicians to presume these diagnoses and initiate appropriate antimicrobial drug treatment (e.g., doxycycline) (*1*), with a successful outcome. Because *R. amblyommatis,* a possibly nonpathogenic species (*1*), was the only agent detected in collected ticks, we consider that the furuncle lesion at the site of the tick bite was a pyogenic, localized, skin infection, and not rickettsia related.

Because of similar clinical manifestations and serologic cross-reactions between typhus group rickettsiae (*1*), we could not confirm the typhus group etiologic agent of this case-patient. We presume *R. typhi* as a probable infection because he had potential occupational exposures with rodents or other small mammals and their ectoparasites, rather than clothing louse infestation (*1*), in addition to an absence of neurologic symptoms, which are more common for epidemic typhus than for murine typhus (*9*,*10*). Conversely, the fact that the patient had spent most of his time in a forest environment could also implicate a sylvatic cycle of typhus group rickettsia in the Amazon forest.

Appendix Additional information on typhus group rickettsiosis, Brazilian Amazon.
