# Supplementary material for: Typhus Group Rickettsiosis, Brazilian Amazon
Source: Emerg Infect Dis. 2020 Sep;26(9):2294–6. doi: 10.3201/eid2609.201305 (PMC7454102; doi:10.3201/eid2609.201305)
Supplement: Appendix — Additional information on typhus group rickettsiosis, Brazilian Amazon. [file 20-1305-Techapp-s1.pdf]

Article DOI: https://doi.org/10.3201/eid2609.201305

Typhus Group Rickettsiosis, Brazilian Amazon

Appendix

Collection and Identification of Ticks in Amazon Forest Area Visited by the Febrile Patient

On October 2019, we collected host-questing ticks by flagging the vegetation in the patient-visited areas in the Saracá-Taquera National Forest, Porto Trombetas District, Brazilian Amazon. In addition, we collected ticks from a fresh carcass of an agouti (*Dasyprocta leporina*) that was found in the area. Collected ticks were transported alive to the laboratory, where they were identified to species according to the procedure of Dantas-Torres et al. (*1*). A total of 170 ticks were identified in 7 species, including 6 *Amblyomma* spp. (Appendix Table).

For molecular detection of rickettsiae, we processed ticks individually (adults and few nymphs) or in pools of 2 to 10 ticks (most of the nymphs) by using the guanidine isothiocyanate phenol technique (*2*) and tested by using PCR and primers CS-78 and CS-323 targeting a 401-bp fragment of the citrate synthase gene, which is common in all representatives of the genus *Rickettsia* (*3*). Samples yielding expected size amplicons were subsequently tested with primers Rr190.70p and Rr190.701n, targeting a 631-bp fragment of the rickettsial 190-kDa outer membrane protein gene, which is present only in members of the spotted fever group (*4*). A negative control tube containing ultrapure water and a positive control tube containing DNA of *Rickettsia vini* were included in each PCR run (*5*). Obtained amplicons were treated with ExoSap (US Biochemicals, https://www.thermofisher.com) and DNA-sequenced in an ABI automated sequencer and model ABI 3500 Genetic Analyzer (Applied Biosystems/Thermo Fisher Scientific, https://www.thermofisher.com) with the same primers used for PCR. Acquired sequences were assembled with Geneious R9 software (https://www.geneious.com) and subjected to blast analyses (https://www.ncbi.nlm.nih.gov) to infer the closest similarities available in GenBank.

The 170 ticks were tested in 50 samples (16 individual adults, 4 individual nymphs and 30 nymphal pools), from which only 17 samples of *A. cajennense* sensu stricto yielded amplicons by both the citrate synthase gene and 190-kDa outer membrane protein gene PCRs. PCR products from these 17 samples yielded DNA sequences that showed 100% identity with *R. amblyommatis* (GenBank accession no. CP012420).

References

1. Dantas-Torres F, Fernandes Martins T, Muñoz-Leal S, Onofrio VC, Barros-Battesti DM. Ticks (Ixodida: Argasidae, Ixodidae) of Brazil: updated species checklist and taxonomic keys. Ticks Tick Borne Dis. 2019;10:101252. PubMed https://doi.org/10.1016/j.ttbdis.2019.06.012

2. Sangioni LA, Horta MC, Vianna MC, Gennari SM, Soares RM, Galvão MA, et al. Rickettsial infection in animals and Brazilian spotted fever endemicity. Emerg Infect Dis. 2005;11:265–70. PubMed https://doi.org/10.3201/eid1102.040656

3. Labruna MB, Whitworth T, Horta MC, Bouyer DH, McBride JW, Pinter A, et al. Rickettsia species infecting *Amblyomma cooperi* ticks from an area in the state of São Paulo, Brazil, where Brazilian spotted fever is endemic. J Clin Microbiol. 2004;42:90–8. PubMed https://doi.org/10.1128/JCM.42.1.90-98.2004

4. Eremeeva ME, Bosserman EA, Demma LJ, Zambrano ML, Blau DM, Dasch GA. Isolation and identification of *Rickettsia massiliae* from *Rhipicephalus sanguineus* ticks collected in Arizona. Appl Environ Microbiol. 2006;72:5569–77. PubMed https://doi.org/10.1128/AEM.00122-06

5. Novakova M, Costa FB, Krause F, Literak I, Labruna MB. *Rickettsia vini* n. sp. (Rickettsiaceae) infecting the tick *Ixodes arboricola* (Acari: Ixodidae). Parasit Vectors. 2016;9:469. PubMed https://doi.org/10.1186/s13071-016-1742-8

Appendix Table. Ticks collected in study of typhus group rickettsiosis, Brazilian Amazon*

Tick species	Source	No. specimens collected	No. samples tested	No. samples yielding rickettsial DNA (% infection)
Amblyomma cajennense sensu stricto	Vegetation	9 adults	9	5 (55)
	Vegetation	100 nymphs	11 pools	11 (11)†
	Agouti	10 nymphs	1 pool	1 (10)†
Amblyomma coelebs	Vegetation	9 nymphs	7 pools	0
Amblyomma naponense	Vegetation	1 adult	1	0
	Vegetation	7 nymphs	3 pools	0
Amblyomma oblongoguttatum	Vegetation	3 adults, 2 nymphs	5	0
Amblyomma pacae	Agouti	2 nymphs	1 pool	0
Amblyomma scalpturatum	Vegetation	3 adults	3	0
	Vegetation	22 nymphs	7 pools	0
Haemaphysalis juxtakochi	Vegetation	2 nymphs	2	0
Total	NA	170	50	17

*NA, not applicable.
†% infection, in this instance, refers to minimal infection rate ($\geq$1 infected tick in each pool): no. infected pools/total no. tested ticks x 100.
